# Dust a Cause of Catarrh

**Published:** 1875-12

**Authors:** 


### Our Exchanges.

---

Dust a Cause of Catarrh.—Dr. W. H. Bennett, of New York, has contributed to the September number of the Medical Record a very able essay upon abnormal conditions of the superior air passages, and some of the causes of chronic inflammation of these parts, in which he ranks dust as one of the most efficient causes. He thinks that he has proved satisfactorily that chronic catarrh may be produced by the action of dust, through experiments upon animals forced to breathe the air which contained no more dust than is frequently seen in the air in certain parts of the business portions of New York. Dust issuing from the registers of houses heated with hot air furnaces, and dust of streets —which is a combination of nearly all substances, filthy and otherwise, known to mankind—he regards as more effective in producing these complaints than clean earth dust and dust occurring in various manufactories. It seems certain that chronic catarrh is more prevalent in cities than in country districts. Dr. Bennett estimates that at least four or five out of every twelve in the city of New York, irrespective of their calling or profession, suffer from chronic inflammation of some part of the respiratory membrane situated above the trachea. Naso-pharyngeal catarrh, he states, is extremely prevalent among the conductors of the city horse-cars, and this disease is always aggravated when the streets are dry, and the consequent amount of dust dragged after the cars is excessive. We think Dr. Bennett is correct in his conclusions, so far as the majority of cases are concerned, and as the amount of dust is unlimited, there seems to be no remedy except to keep out of it, and keep the nasal passages as clean as possible. *Herald of Health.*

